# The prognostic and immunological role of MCM3 in pan-cancer and validation of prognosis in a clinical lower-grade glioma cohort

**DOI:** 10.3389/fphar.2024.1390615

**Published:** 2024-04-18

**Authors:** Qian-Rong Huang, Qian Jiang, Ju-Yuan Tan, Ren-Bao Nong, Jun Yan, Xia-Wei Yang, Li-Gen Mo, Guo-Yuan Ling, Teng Deng, Yi-Zhen Gong

**Affiliations:** ^1^ Department of Neurosurgery, Guangxi Medical University Cancer Hospital, Nanning, China; ^2^ Guangxi Medical University, Nanning, China; ^3^ Department of Clinical Research, Guangxi Medical University Cancer Hospital, Nanning, China

**Keywords:** pan-cancer, minichromosome maintenance complex component 3 (MCM3), immunotherapy, prognosis, lower-grade glioma

## Abstract

**Background:** Previous studies have shown that MCM3 plays a key role in initiating DNA replication. However, the mechanism of MCM3 function in most cancers is still unknown. The aim of our study was to explore the expression, prognostic role, and immunological characteristics of MCM3 across cancers.

**Methods:** We explored the expression pattern of MCM3 across cancers. We subsequently explored the prognostic value of MCM3 expression by using univariate Cox regression analysis. Spearman correlation analysis was performed to determine the correlations between MCM3 and immune-related characteristics, mismatching repair (MMR) signatures, RNA modulator genes, cancer stemness, programmed cell death (PCD) gene expression, tumour mutation burden (TMB), microsatellite instability (MSI), and neoantigen levels. The role of MCM3 in predicting the response to immune checkpoint blockade (ICB) therapy was further evaluated in four immunotherapy cohorts. Single-cell data from CancerSEA were analysed to assess the biological functions associated with MCM3 in 14 cancers. The clinical correlation and independent prognostic significance of MCM3 were further analysed in the TCGA and CGGA lower-grade glioma (LGG) cohorts, and a prognostic nomogram was constructed. Immunohistochemistry in a clinical cohort was utilized to validate the prognostic utility of MCM3 expression in LGG.

**Results:** MCM3 expression was upregulated in most tumours and strongly associated with patient outcomes in many cancers. Correlation analyses demonstrated that MCM3 expression was closely linked to immune cell infiltration, immune checkpoints, MMR genes, RNA modulator genes, cancer stemness, PCD genes and the TMB in most tumours. There was an obvious difference in outcomes between patients with high MCM3 expression and those with low MCM3 expression in the 4 ICB treatment cohorts. Single-cell analysis indicated that MCM3 was mainly linked to the cell cycle, DNA damage and DNA repair. The expression of MCM3 was associated with the clinical features of LGG patients and was an independent prognostic indicator. Finally, the prognostic significance of MCM3 in LGG was validated in a clinical cohort.

**Conclusion:** Our study suggested that MCM3 can be used as a potential prognostic marker for cancers and may be associated with tumour immunity. In addition, MCM3 is a promising predictor of immunotherapy responses.

## Introduction

In recent decades, the incidence of cancer has been increasing, which has been fuelled by population growth, population ageing, and the implementation of advanced early detection and treatment modalities. This scenario has led to an ever-growing population of cancer survivors, thus contributing to an alarming global burden of cancer that now represents a significant threat to the health of humanity (Sung et al., 2021). Despite the rapid development of early prevention and treatment techniques for cancer, the mortality rate of cancer remains a concern (Torre et al., 2015; Miller et al., 2022). As research continues to advance, an increasing number of researchers are focusing on the common features of cancer. Pan-cancer analysis, which compares data such as gene mutation, gene expression, and protein expression data across different types of cancer, employs sophisticated bioinformatics techniques to identify shared and distinct characteristics among cancers (Cancer Genome Atlas Research et al., 2013). Previous research has demonstrated that a number of genes are crucial for the immune microenvironment, prognosis, and drug resistance in pan-cancer (Fu et al., 2022; Pan et al., 2022; Xie et al., 2022; Zhou et al., 2023). Consequently, this analytical approach has proven to be a powerful tool for investigating the genetic and molecular basis of numerous cancer types (Xu et al., 2020; Chen et al., 2021).

Minichromosome maintenance complex component 3 (MCM3) is a crucial member of the MCM protein family that interacts with other members (MCM2 and MCM4-7) to form a durable heterohexameric complex, which plays a pivotal role in initiating DNA replication (Madine et al., 2000; Evrin et al., 2014; Deegan and Diffley, 2016; Sedlackova et al., 2020). MCM3 is highly expressed in diverse types of malignancies, including breast cancer (Lokkegaard et al., 2021), ovarian cancer (Li et al., 2021), colorectal cancer (Zhou et al., 2020), and prostate cancer (Hsu et al., 2021). Notably, multiple studies have demonstrated that elevated MCM3 expression is strongly linked to tumour progression, metastasis, and prognosis (Li et al., 2021; Lokkegaard et al., 2021; Yang et al., 2019; Zhou et al., 2020). In addition, phosphorylated MCM3 has been shown to promote cell proliferation and inhibit cell apoptosis in renal cell carcinoma cells (Gao et al., 2022). Another study suggested that the MCM3 proliferation index was more clinically relevant than Ki-67 in the characterization of salivary gland tumours (Raja et al., 2021). Indeed, MCM3 not only plays a crucial role in DNA replication but is also involved in the DNA damage response and DNA repair (Drissi et al., 2018). Cancers rely on the activation of DNA repair pathways to maintain genomic stability, stemness, and chemotherapy resistance (Abad et al., 2020; Wang et al., 2021); therefore, further evaluation of the relationship of MCM3 with DNA repair genes and cancer stemness across cancers is warranted. Overall, investigations of MCM3 have been largely limited to a small number of cancer types, and the role of MCM3 in various malignancies and the underlying mechanisms remain incompletely understood.

Herein, we comprehensively assessed the expression and prognostic role of MCM3 across cancers. We then systematically evaluated the associations of MCM3 with immune signatures, mismatching repair (MMR) genes, RNA modulator genes, cancer stemness, programmed cell death (PCD) genes, the TMB, MSI, and neoantigen levels. In our study, the ability of MCM3 to predict immunotherapy response was also evaluated, and its association with 14 biological functions was evaluated at the single-cell level. In addition, based on data from The Cancer Genome Atlas (TCGA) and Chinese Glioma Genome Atlas (CGGA) databases, further clinical correlation analysis, independent prognostic analysis, nomogram construction, and biological function exploration were conducted in LGG cohorts. Finally, the clinical correlation and prognostic significance of MCM3 in LGG were verified in a clinical cohort from Guangxi Medical University Cancer Hospital.

## Materials and methods

### Data collection and expression analysis

The mRNA data (TPM) for the TCGA pan-cancer cohort and corresponding normal tissues from the GTEx were downloaded from UCSC database. Survival data for each type of cancer were also downloaded from UCSC. The mRNA data were log2 (TPM+1) transformed. We first compared MCM3 mRNA expression in tumour and normal tissues and evaluated the differences between groups by using the Wilcoxon test. We further evaluated MCM3 protein levels across cancers by utilizing the CPTAC portal in the UALCAN database. By employing the GEPIA online database, we preliminarily investigated the relationship between MCM3 expression and clinical stage. In this study, we also explored the genomic alterations of MCM3 across cancers by using the cBioPortal database. Immunofluorescence (IF) images from the HPA database were used to identify the subcellular localization of MCM3 in tumours. In addition, immunohistochemistry (IHC) was used to compare MCM3 protein expression in LGG and normal brain tissue.

### Prognosis evaluation

We analysed the association between MCM3 expression and patient outcomes for each cancer type. In this study, we evaluated four prognostic indicators, including overall survival (OS), progression-free interval (PFI), disease-specific survival (DSS), and disease-free interval (DFI). MCM3 expression was included as a continuous variable in univariate Cox regression analysis according to the “survival” package in R. In addition, a heatmap was generated to display the survival analysis results associated with MCM3 across cancers.

### Assessment of relevant characteristics

The tumour microenvironment (TME) plays a key role in tumour formation and progression (Xiao 2021; Zou et al., 2023). We assessed the relationships between MCM3 and TME-related parameters (immune, stromal and ESTIMATE scores) across cancers by using the “estimate” package (Yoshihara et al., 2013). By using the “IOBR” package, we applied the TIMER algorithm to measure the relationship between MCM3 expression and the infiltration levels of six immune cell types across cancers (Li et al., 2017). We extracted expression data for immune checkpoint components (inhibitory and stimulatory) and several PCD (pyroptosis, cuproptosis, anoikis, necroptosis, disulfdptosis and autophagy) markers and evaluated the association of MCM3 expression with these markers. The correlation of MCM3 with 5 MMR signatures and 44 RNA modification genes (m1A, m5C and m6A) in pan-cancer was analysed (Liang et al., 2022). In addition, we obtained pan-cancer differentially methylated probe-based stemness index (DMPsi) from the study by Malta et al. to determine the relationship between MCM3 expression and cancer stemness (Malta et al., 2018).

### Immunotherapy prediction and drug sensitivity analysis

TMB, MSI, and neoantigens have been reported to influence cancer prognosis and immunotherapy response (Ettinger et al., 2019; Picard et al., 2020). We also investigated the association of MCM3 with these markers. In addition, we selected four cohorts of tumour patients who were receiving ICB therapy to further evaluate the ability of MCM3 to predict the response to immunotherapy. By using the “survminer” package in R, we determined the optimal cut-off value and divided the IMvigor210 (urinary tumours), GSE176307 (urothelial cancer), GSE135222 (non-small cell lung cancer, NSCLC) and GSE91061 (melanoma) cohorts into high-MCM3 and low-MCM3 groups. We then compared the outcomes and treatment responses between the high-MCM3 and low-MCM3 subgroups. The data for these immunotherapy cohorts were obtained from the http://research-pub.gene.com/IMvigor210CoreBiologies/packageVersions/, TIGER (http://tiger.canceromics.org/#/download) and GEO (https://www.ncbi.nlm.nih.gov/geo/) websites. Finally, we explored the correlation between MCM3 expression and drug sensitivity by using CTRP and GDSC data from the GSCA database (http://bioinfo.life.hust.edu.cn/GSCA/#/drug) to help identify potential drugs targeting MCM3.

### Single-cell analysis

CancerSEA is a single-cell sequencing database for assessing the status and function of single cells in a variety of tumours (Yuan et al., 2019). Herein, we analysed the correlation between MCM3 expression and 14 functions based on single-cell data from the CancerSEA database via Spearman analysis. In addition, by using single-cell data from the TISCH database (http://tisch.comp-genomics.org/) (Sun et al., 2021), we further evaluated the expression of MCM3 in different cancer cell subtypes.

### Clinical correlation analysis of MCM3 in LGG

For in-depth analysis of LGG, mRNA data (FPKM) and clinical information from LGG cohorts were obtained from the TCGA and CGGA databases. We transformed the expression data *via* log2(FPKM+1) transformation. We then analysed the relationships between MCM3 mRNA expression and five clinical parameters, and the differences between the two groups were evaluated by using the Wilcoxon test. Based on the median expression value, we compared the OS of the high-MCM3 and low-MCM3 expression groups by using the Kaplan–Meier (KM) method. A time-dependent receiver operating characteristic (timeROC) curve was used to evaluate the efficacy of MCM3 in predicting survival. These analyses were performed by using the “timeROC” package in R. By using three glioma single-cell datasets (GSE139448, GSE163108 and GSE162631) in the TISCH database, MCM3 expression in different immune cells of glioma was evaluated.

### Nomogram construction, enrichment analysis and TMB analysis in LGG

The prognostic role of MCM3 was further assessed via multivariate analysis in the TCGA training cohort. With the “rms” package, independent prognostic features were selected to construct a prediction nomogram. The CGGA cohort served as the validation cohort. We used timeROC curves, calibration curves, decision curve analysis (DCA) and Kaplan–Meier (KM) curves to systematically evaluate the predictive ability of the model. To explore more potential biological mechanisms of MCM3 activity in LGG, differentially expressed genes (DEGs) between the two subgroups of the TCGA cohort were identified, and enrichment analyses, including GO, KEGG, and GSEA, were performed by using the “clusterProfiler” and “enrichplot” packages. P. adjust <0.05 was used as the significance threshold for enrichment analysis in GO and KEGG analyses, while *p* < 0.05 was defined as statistically significant in GESA. We also analysed the mutation frequency and TMB of the two subgroups in the TCGA cohort. The mutation data were evaluated and visualized by using the “maftools” software package. The TIDE algorithm is a novel tool for evaluating immunotherapy responses (Cao et al., 2020). We calculated TIDE scores for the TCGA-LGG cohort by using the TIDE database and compared the scores between the two subgroups.

### Validation of the prognostic significance of MCM3 in LGG

We first compared MCM3 protein levels in LGG and normal brain tissue by using the HPA database. We then validated the prognostic value of MCM3 in LGG in a clinical cohort. Tumour specimens and clinicopathological parameters were collected from patients with newly diagnosed LGG who underwent surgical treatment at Guangxi Medical University Cancer Hospital between May 2013 and December 2018. Tumour specimens were embedded in paraffin immediately after collection, and clinicopathological information was collected for all of the patients. This clinical cohort was followed up until July 2019, with death or progression as the end events, and both OS and progression-free survival (PFS) were calculated. IHC staining for MCM3 was then performed on paraffin-embedded LGG tissue. The MCM3 antibody was purchased from Boster Biological Technology Company (article number: BA2186). The percentage of positively stained cells was scored as follows: 0 (0%), 1 (1%–25%), 2 (26%–50%), 3 (51%–75%), and 4 (76%–100%). The intensity of staining was scored as follows: 0 (no staining), 1 (weak), 2 (moderate), and 3 (strong). The expression of MCM3 was determined as the product of these two scores. Ultimately, a score of 0–2 was defined as indicating negative MCM3 expression, and a score of 3–12 was defined as indicating positive MCM3 expression (Zhang et al., 2016).

### Statistical analysis

In this study, comparisons of MCM3 expression in normal and tumour tissues and analyses of the correlation of MCM3 levels with clinical features in LGG were performed via the Wilcoxon test. The prognostic significance of MCM3 was assessed by using univariate, multivariate Cox and Kaplan–Meier (KM) (log-rank test) analyses. The relationships between MCM3 expression and immunological characteristics, PCD gene expression, TMB, MSI, neoantigen levels, and biological functions at the single-cell level were evaluated via Spearman analysis. Chi-square tests were utilized to compare the proportions between two groups. The remaining methods are described in the Methods section. A P-value <0.05 was considered to indicate statistical significance. The analysis and graphing in this study were performed in R (v 3.6.3). A portion of the pan-cancer analysis and graphing of MCM3 was performed through two online websites (Home for researchers [https://www.home-for-researchers.com/static/index.html#/] and Xiantaoxueshu [https://www.xiantaozi.com/]).

## Results

### Expression patterns of MCM3 across cancers

**FIGURE 1:**
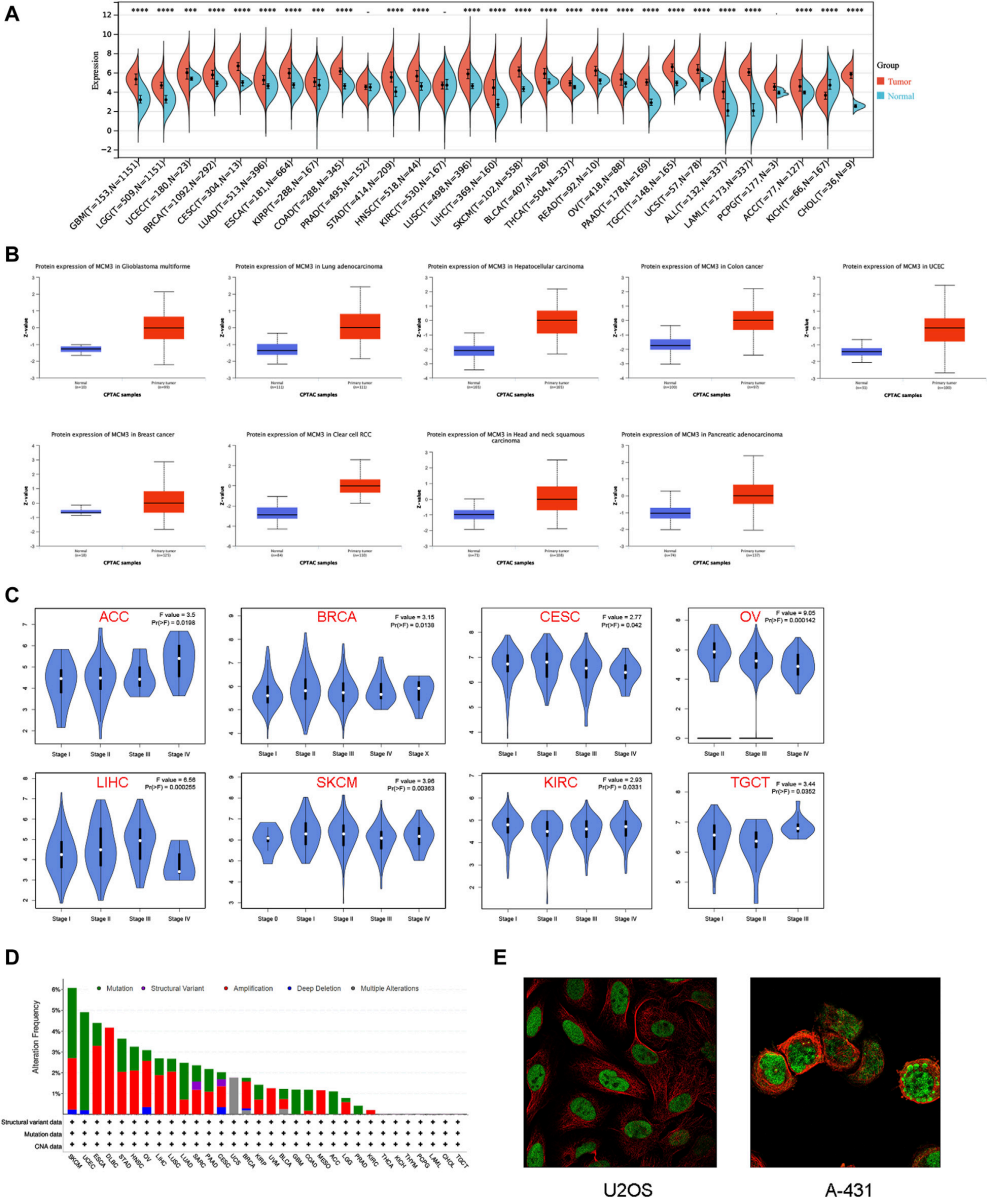
Expression patterns of MCM3 in pan-cancer. **(A)** Differences in MCM3 between tumor and normal tissues based on TCGA and GETx data. **(B)** Comparison of protein levels based on CPTAC data. **(C)** Clinical correlation analysis based on GEPIA database. **(D)** The genomic alteration of MCM3 in pan-cancer. **(E)** Immunofluorescence results showed the localization of MCM3 in cell lines. ****p < 0.0001.

We first investigated the differences in MCM3 mRNA levels between normal and tumour tissues. The results showed that the expression of MCM3 was upregulated in most tumours but significantly lower in KICH tissues than in normal tissues. In PRAD, KIRC, and PCPG, the differences were not significant (Figure 1A). Data from the CPTAC portal confirmed elevated MCM3 protein expression in a variety of cancers, including GBM, LUAD, LIHC, COAD, UCEC, BRCA, KIRC, HNSC, and PAAD (Figure 1B). By using the GEPIA database, we analysed the association between MCM3 and clinical stage across cancers. We found that MCM3 expression was significantly associated with the clinical stages of eight cancers, including ACC, BRCA, CESC, KIRC, LIHC, OV, SKCM and TGCT (Figure 1C). We further explored the genomic alteration status of MCM3 across cancers via the cBioPortal website. Overall, genetic variations in MCM3 occur in less than 5% of most cancers. The highest frequency of MCM3 variants (>6%) was found in SKCM, with “mutation” and “amplification” being the main types. UCEC had the highest incidence (>4%) of “mutations”, whereas DLBC had the highest incidence (>4%) of “amplification” (Figure 1D). IF of tumour cells from the HPA database showed that MCM3 protein was localized in the nuclei of U2OS (osteosarcoma) and A-431 (cutaneous squamous cell) cell lines (Figure 1E).

### Prognostic significance of MCM3 across cancers

**FIGURE 2:**
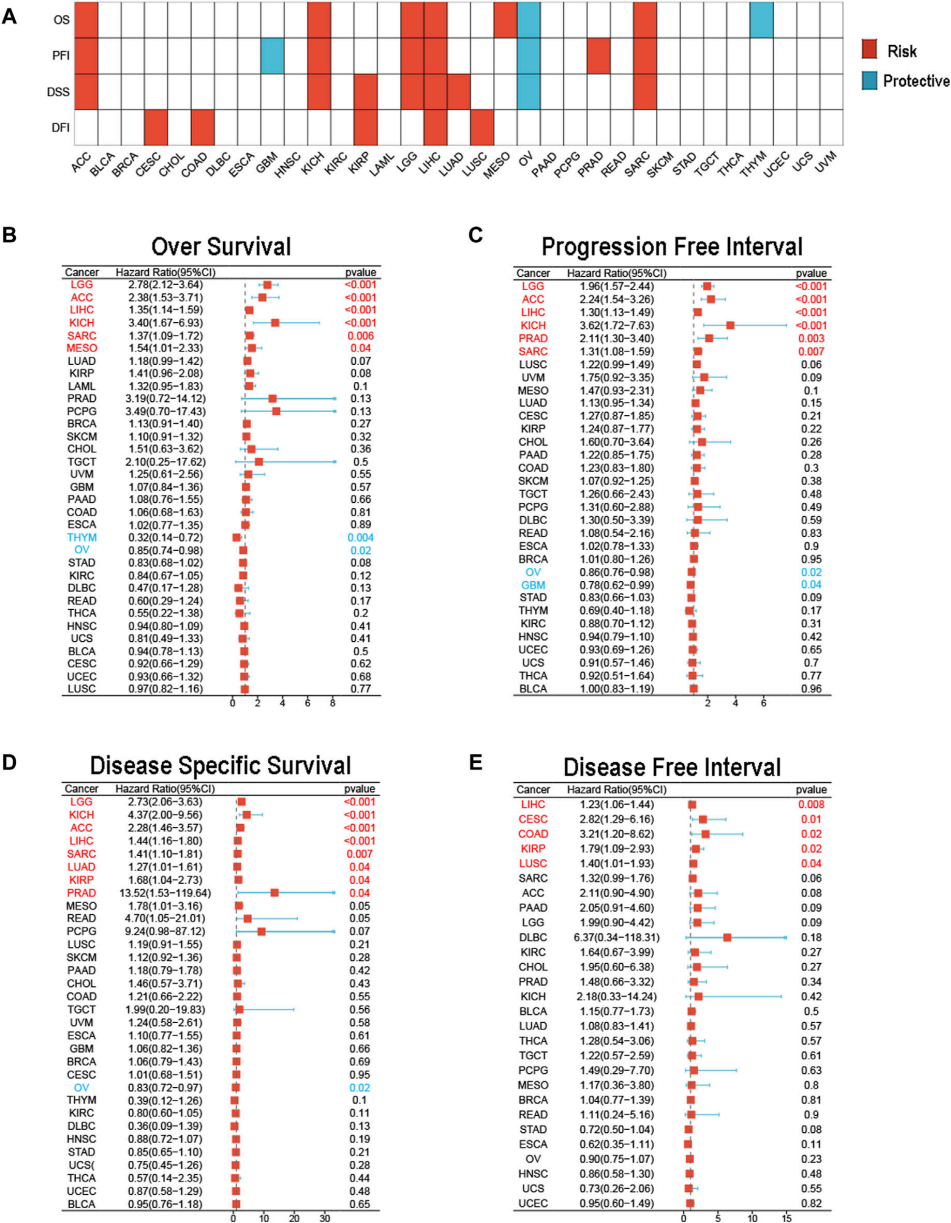
Prognostic value of MCM3 in pan-cancer. **(A)** The heatmap shows results of univariate Cox regression analysis. **(B)** Forest plot of MCM3 expression and OS across cancers. **(C)** Forest plot of MCM3 expression and PFI across cancers. **(D)** Forest plot of MCM3 expression and DSS across cancers. **(E)** Forest plot of MCM3 expression and DFI across cancers.

We subsequently used univariate Cox analysis to explore the prognostic significance of MCM3 in multiple aspects, including OS, the PFI, DSS and the DFI. The results showed that MCM3 expression levels were closely linked to different outcomes in many cancers (Figure 2A). For OS, MCM3 was a risk index for LGG, ACC, LIHC, KICH, SARC and MESO but a protective factor for OV and THYM (Figure 2B). The upregulation of MCM3 suggested that LGG, ACC, LIHC, KICH, PRAD and SARC had shorter PFIs, whereas OV and GBM had longer PFIs (Figure 2C). For DSS, MCM3 expression was a risk factor in LGG, KICH, ACC, LIHC, SARC, LUAD, KIRP and PRAD and a protective factor in OV (Figure 2D). In addition, high MCM3 expression was associated with a shorter DFI in LIHC, CESC, COAD, KIRP and LUSC (Figure 2E).

### Association between MCM3 expression and tumour immunity

**FIGURE 3:**
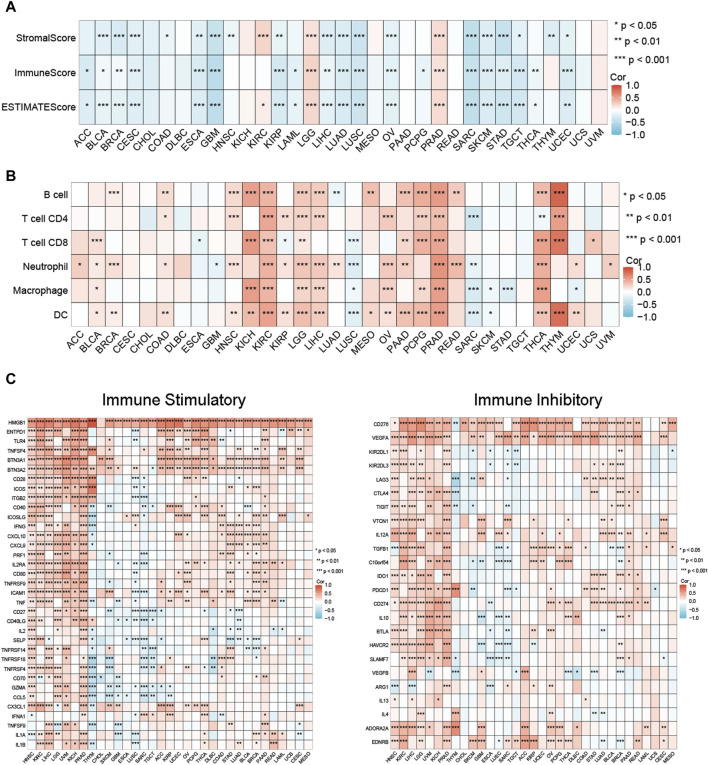
Relationship between MCM expression and immune-related features in pan-cancer. **(A)** MCM3 expression and tumor microenvironment relate parameters. **(B)** MCM3 expression and immune cell infiltration. **(C)** MCM3 expression and immune checkpoints. *p < 0.05, **p < 0.01, ***p < 0.001.

We investigated the relationship between MCM3 and tumour immunity in three aspects, including the TME profile, immune cell infiltration and immune checkpoint factor expression. The results showed that MCM3 expression was negatively correlated with the stromal score, immune score, and ESTIMATE score in most tumours, whereas significant positive correlations with these scores were observed in LGG, KIRC and PRAD (Figure 3A). By using the TIMER algorithm, we evaluated the infiltration levels of six immune cell types across cancers. MCM3 expression was positively correlated with immune cell infiltration in most tumours, especially in KIRC, LGG, LIHC, PCPG, PRAD and THCA (Figure 3B). Similarly, MCM3 expression was positively correlated with immune checkpoint factor expression in most tumours, especially in HNSC, KIRC, LIHC, LGG, UVM, KICH, and PRAD (Figure 3C). Surprisingly, MCM3 was positively correlated with the expression of several immune checkpoint molecules, such as HMGB1, BTN3A2, CD276, and VEGFA, in almost all of the tumours.

For the MMR signatures, we found a significant positive correlation between them and MCM3 broadly in pan-cancer, and a negative correlation between EPCAM and MCM3 expression in LGG and THYM (Supplementary Figure S1A). Moreover, we observed a positive correlation between MCM3 expression and DMPsi in DLBC, LGG and STAD and a negative correlation between MCM3 expression and THYM, KIRP and THCA (Supplementary Figure S1B), thus suggesting that MCM3 may be involved in DNA repair-mediated cancer stemness. Interestingly, we also found that MCM3 was positively correlated with most RNA modulator genes across cancers (Supplementary Figure S1C).

Recently, PCD modalities, such as pyroptosis, cuproptosis, anoikis, necroptosis, disulfdptosis and autophagy, have been reported to play important roles in the development and progression of cancer. Therefore, we further explored the relationship between MCM3 and markers of PCD. The results indicated a general correlation between MCM3 and PCD markers. Among these genes, MCM3 was significantly correlated with most pyroptosis genes across cancers, except for CHOL, MESO and UCS (Supplementary Figure S2A). Similarly, MCM3 was generally associated with markers of cuproptosis, anoikis, necroptosis, disulfdptosis and autophagy in most tumours (Supplementary Figure S2B–F).

### Associations of MCM3 with immunotherapy response and drug sensitivity

**FIGURE 4:**
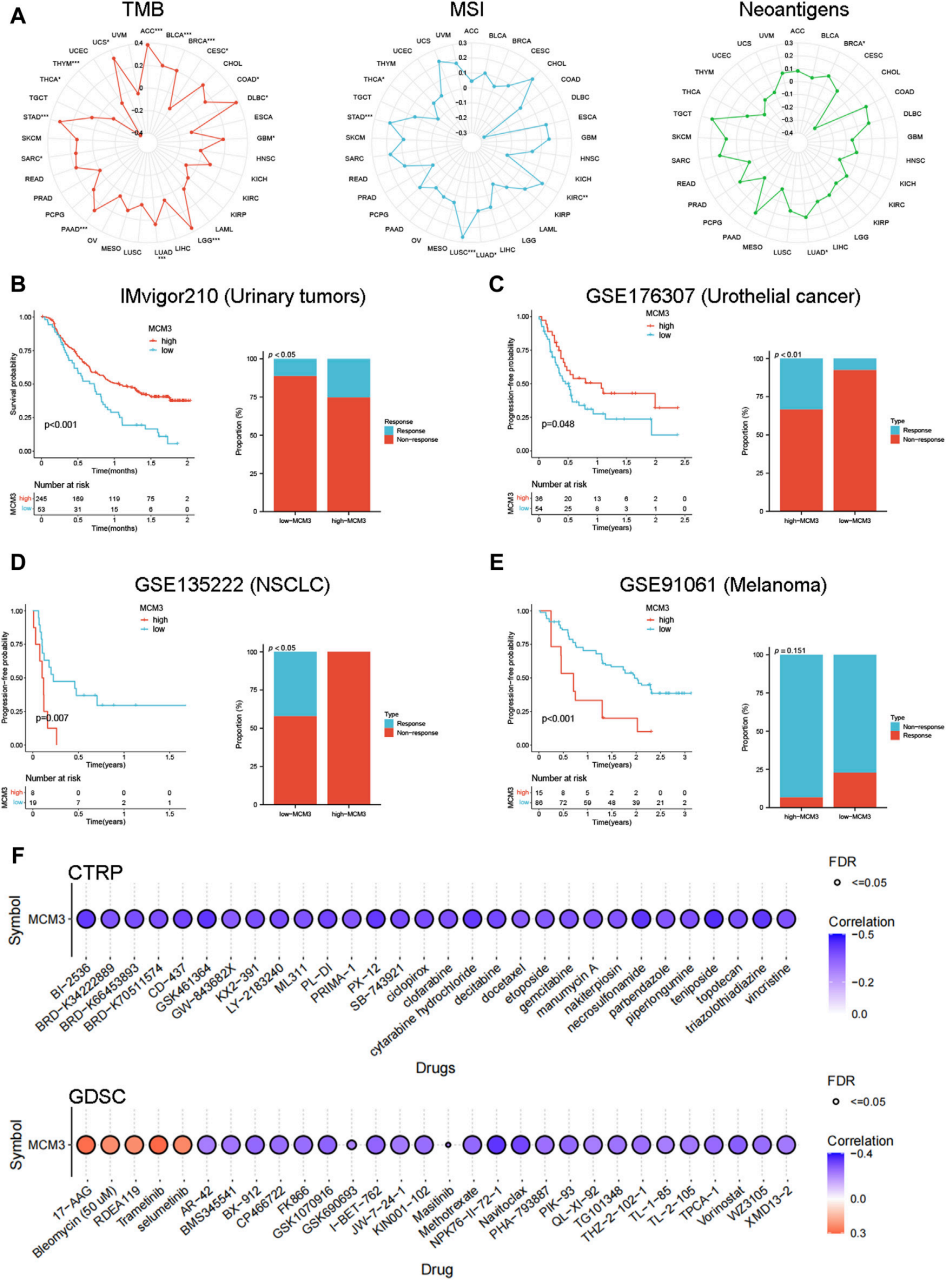
Immunotherapy and drug sensitivity analysis. **(A)** Relationship between MCM3 expression and TMB, MSI, and neoantigens. **(B–E)** Prognostic significance of MCM3 and proportion of immunotherapy response between high- and low-MCM3 groups in four cohorts receiving ICB therapy. **(F)** Drug sensitivity analysis of MCM3 based on CTPR and GDSC data. *p < 0.05, **p < 0.01, ***p < 0.001.

Considering that TMB, MSI, and neoantigen expression are common genomic alterations that are closely associated with cancer prognosis and immunotherapeutic responses (Ettinger et al., 2019; Ben-David and Amon, 2020; Picard et al., 2020), we measured the association between MCM3 and these alterations across cancers. As shown in Figure 4A, MCM3 and TMB were positively correlated in 12 cancers and negatively correlated in three cancers. There was a positive correlation between MCM3 expression and MSI in KIRC, LUAD, LUSC and STAD but a negative correlation in THCA. In contrast, the correlation of neoantigen expression with MCM3 was generally not significant; additionally, a positive correlation was shown only in BRCA and LUAD (Figure 4A). We subsequently evaluated the ability of MCM3 to predict the response to immunotherapy in four clinical cohorts receiving ICB therapy. Survival analysis demonstrated an obvious difference in prognosis between the two subgroups in all of the cohorts (Figures 4B–E). In addition to the GSE91061 cohort, there were significant differences in the proportion of patients who experienced treatment benefits between the two subgroups (Figures 4B–E). These data indicated that MCM3 expression could effectively distinguish patients who had different responses to ICB treatment and further suggested that MCM3 could be used as a potential marker to assess immunotherapy responses. Finally, based on data from the CTRP and GDSC databases, we investigated the association of MCM3 with drug sensitivity to explore potential targeted drugs. The CTRP results demonstrated that the MCM3 level was negatively correlated with sensitivity to most drugs. The GDSC results showed that sensitivity to five drugs, including 17-AAG, bleomycin (50 µM), RDEA119, trametinib and selumetinib, was positively correlated with MCM3 expression, whereas sensitivity to other drugs was negatively correlated with MCM3 expression (Figure 4F). These results may provide a basis for developing therapies targeting MCM3.

### Single-cell analysis

**FIGURE 5:**
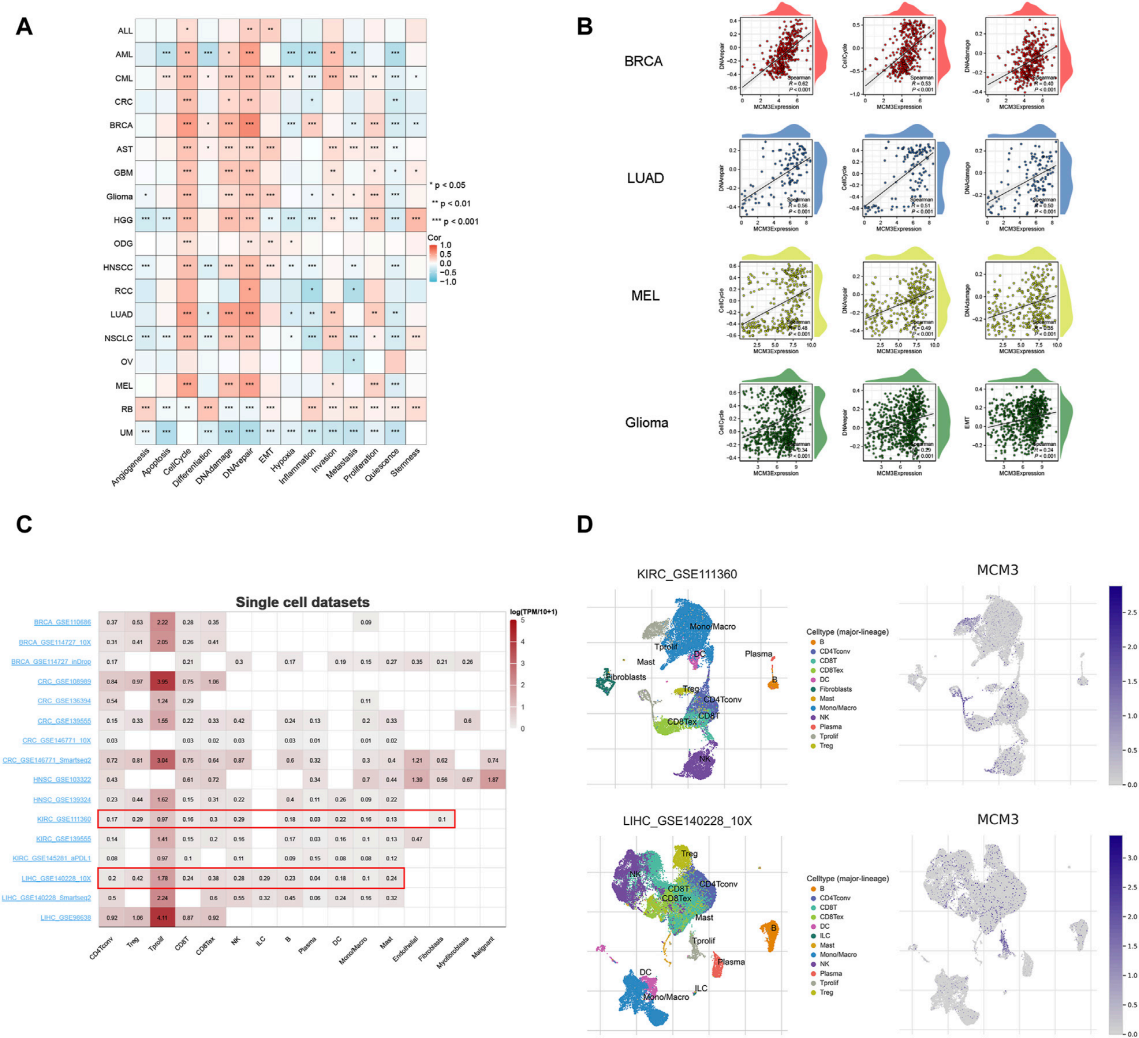
Single-cell analysis of MCM3. **(A)** Correlation between MCM3 and 14 biological functions. **(B)** The top three functions in BRCA, LUAD, MEL and glioma. **(C)** Datasets of single-cell expression of MCM3 from TISCH website. **(D)** Distribution of MCM3 among cell types in the GSE111360 and GSE140228 datasets. *p < 0.05, **p < 0.01, ***p < 0.001.

We evaluated the relationship between MCM3 and 14 biological functions in multiple cancers at the single-cell level by using the CancerSEA database. As the heatmap shows, MCM3 is closely linked to these biological functions in most cancers. Among them, the cell cycle, DNA damage and DNA repair had the most significant correlations with MCM3 expression (Figure 5A). In addition, we generated correlation plots of the top three functions in BRCA, LUAD, MEL, and glioma (Figure 5B). The TISCH data showed that MCM3 was widely expressed in most immune cells, with major concentrations in CD4Tconv, Treg, Tprolif, CD8T, CD8Tex and NK cells (Figure 5C). Figure 5D shows the expression of MCM3 in the GSE111360 and GSE140228 single-cell datasets collected.

### Clinical correlation analysis of MCM3 in LGG

**FIGURE 6:**
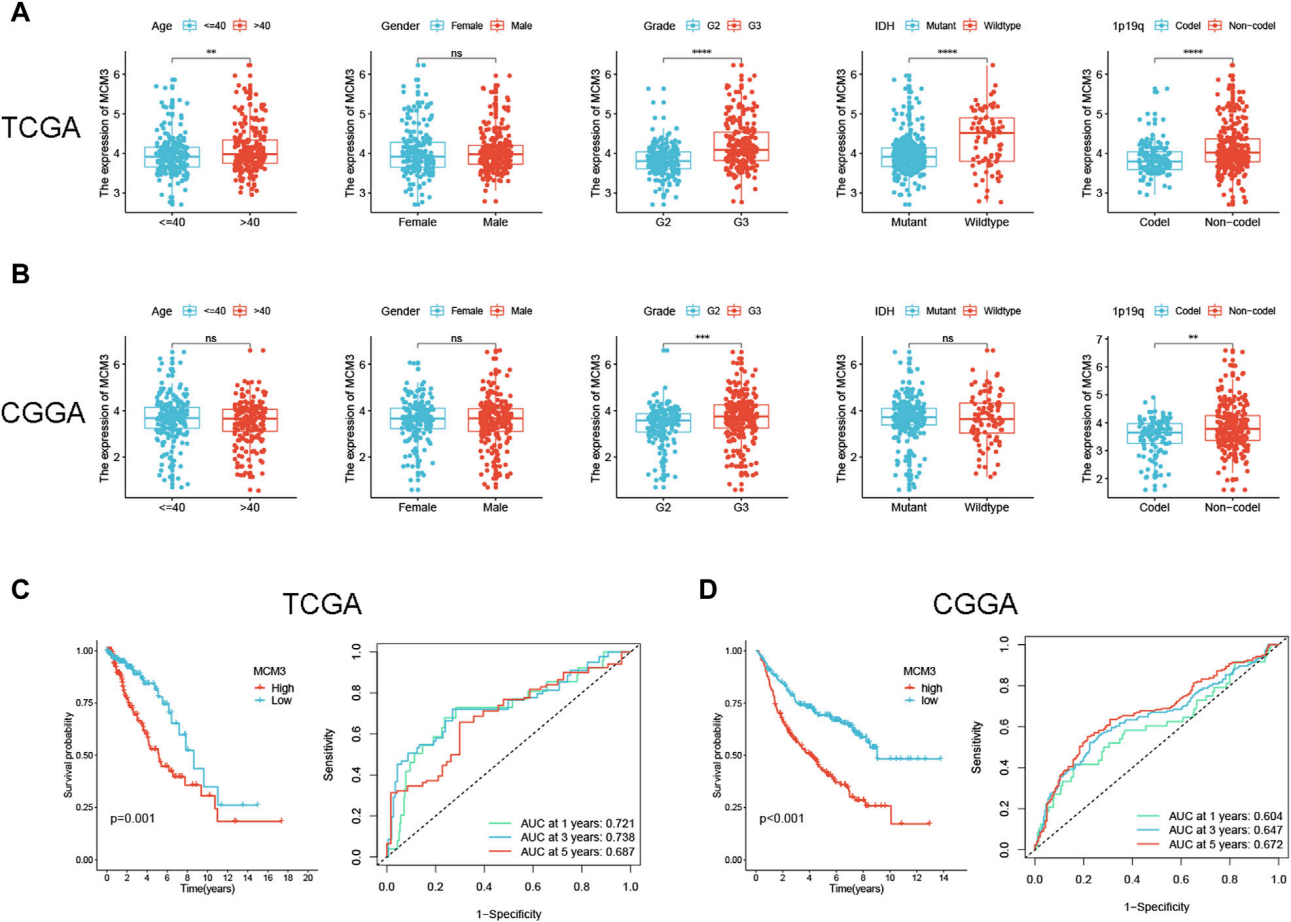
Correlation between MCM3 expression and clinical features and prognosis of LGG. **(A, B)** Relationship between MCM3 and clinical features in TCGA and CGGA cohorts. **(C, D)** Evaluation of the ability of MCM3 expression to predict prognosis in TCGA and CGGA cohorts. ns: no significance, **p < 0.01, ***p < 0.001, ****p < 0.0001.

Preliminary results indicated that MCM3 was dysregulated in LGG and could be used as a prognostic predictor of LGG (including OS, PFI and DSS). In addition, MCM3 expression was closely related to various immune features of LGG. We further investigated the correlation between MCM3 and clinical parameters and verified its prognostic value in LGG. The clinical data of the two publicly available cohorts are shown in Supplementary Table S1. In the TCGA cohort, MCM3 expression was closely correlated with age, grade, IDH expression, and 1p19q deletion status (Figure 6A). In CGGA cohort, MCM3 expression was closely linked to the grade and 1p19q deletion status (Figure 6B). KM analysis also showed that MCM3 has good predictive value. In the TCGA cohort, patients with low MCM3 expression had longer OS than those with high MCM3 expression, and the AUC values of MCM3 expression for predicting 1-, 3- and 5-year survival in LGG patients were 0.721, 0.738 and 0.687, respectively. (Figure 6C). The KM curve of the CGGA cohort showed similar results, with AUC values of 0.604, 0.647 and 0.672, respectively (Figure 6D). We evaluated MCM3 expression in immune cells by using three datasets from the TISCH database. In the GSE139448 dataset, MCM3 expression was highest in malignant cells, whereas in the GSE163108 and GSE162631 datasets, MCM3 expression was highest in Tprolif and Mono/Macro cells, respectively (Supplementary Figure S3). Our findings demonstrated that the expression of MCM3 differed in different cell types and that there were differences in cell components among samples, which may be related to the heterogeneity of the glioma microenvironment.

### Nomogram construction, enrichment analysis and TMB analysis in LGG

**FIGURE 7:**
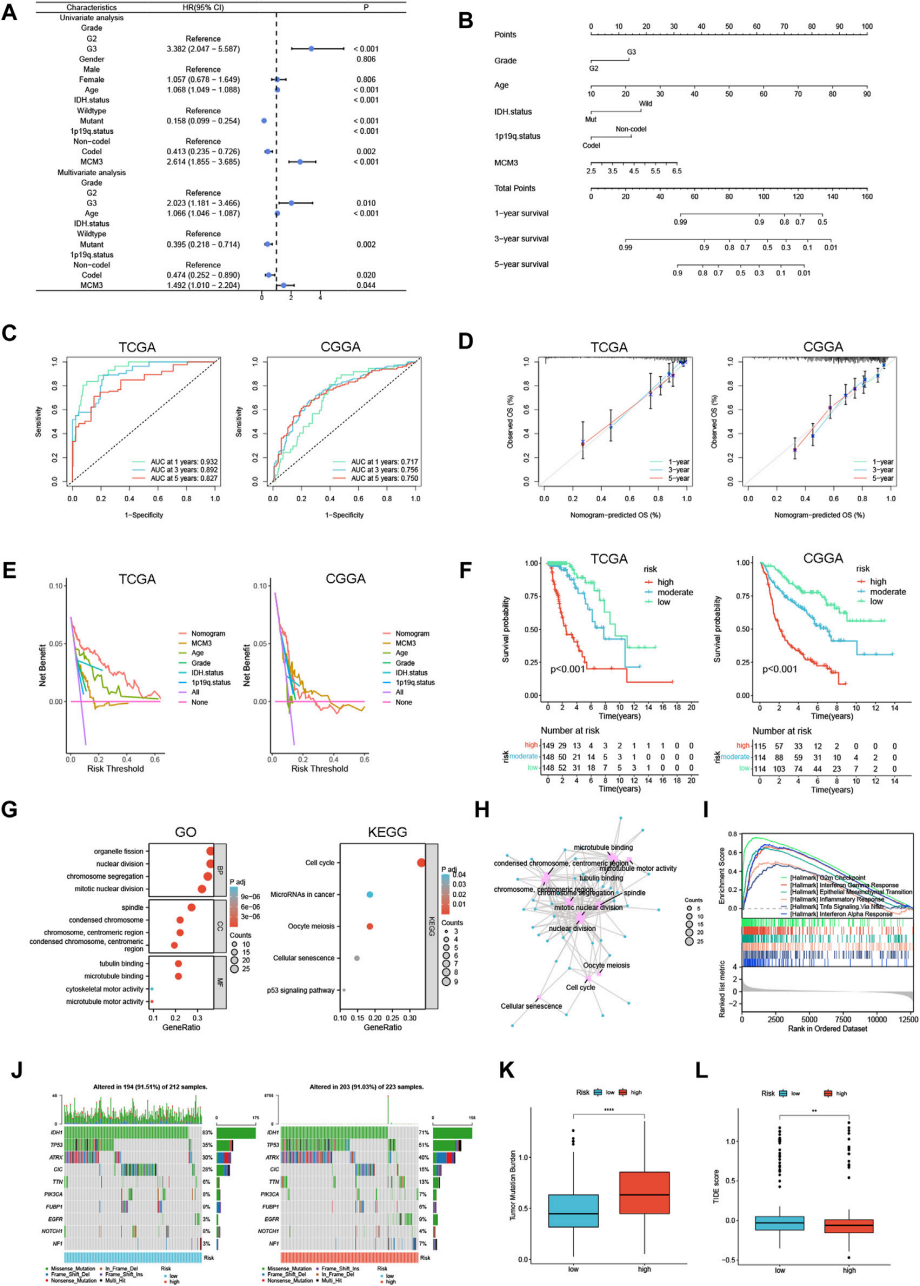
Construction of a prognostic nomogram and analysis of MCM3 related functions in LGG. **(A)** Univariate/multivariate Cox analysis was performed based on TCGA cohort. **(B)** Establishment of a prognostic nomogram based on multivariate analysis results. **(C–F)** Nomogram model evaluation, including timeROC, calibration, DCA and KM curves. **(G)** GO and KEGG analyses based on differentially expressed genes in TCGA. **(H)** An interaction network between GO and KEGG. **(I)** The GSEA analysis in TCGA cohort. **(J)** The waterfall map shows the top 10 genes with the highest mutation probability. **(K, L)** TMB and TIDE score were compared between the two groups. **p < 0.01, ****p < 0.0001.

Furthermore, univariate/multivariate Cox analysis indicated that MCM3 had independent prognostic significance in the TCGA cohort (Figure 7A). To improve the clinical application value of MCM3, our study incorporated independent prognostic parameters from the TCGA cohort to construct a nomogram, and the CGGA cohort served as the validation cohort (Figure 7B). The model AUC values were significantly improved in both cohorts (Figure 7C). The calibration curves showed that the results predicted by the model were close to the actual observed results (Figure 7D). The DCA curves showed that the model was more beneficial for predicting the outcome of LGG patients than any single prognostic factor (Figure 7E). Based on the risk scores calculated by the model, the two cohorts were evenly divided into three subgroups, and the survival analysis indicated obvious differences in OS among the subgroups (Figure 7F), which further suggested that the MCM3-based prognostic nomogram could be an effective risk assessment tool for LGG patients. A total of 77 DEGs between the high-MCM3 and low-MCM3 groups in the TCGA training cohort were identified. GO results indicated that the DEGs were closely linked to organelle fission, nuclear division, chromosome segregation, mitotic nuclear division, chromosomes, centromeric region and microtubule binding. According to the KEGG analysis, these DEGs were mainly enriched in the terms “cell cycle”, “microRNAs in cancer”, “cellular senescence” and “p53 signalling pathway” (Figure 7G). An interactive network plot was constructed to show the relationships between the GO and KEGG terms (Figure 7H). We also performed GSEA, and several common functions and pathways, such as the G2M checkpoint, epithelial mesenchymal transition (EMT), inflammatory response, Tnfa signalling *via* Nfkb, and interferon alpha response, were obviously enriched in the TCGA high-MCM3 group (NES>1.5, *p*-value <0.001) (Figure 7I). The waterfall map shows the top 10 genes with the highest mutation probability shared by the two subgroups (Figure 7J). Notably, IDH1 has been identified as being an important prognostic marker for LGG, and the likelihood of IDH mutation is greater in the low-MCM3 subgroup, which to some extent explains the better prognosis in this subgroup. In addition, we investigated the relationships of MCM3 with TMB and TIDE, and our data demonstrated that the high-MCM3 group had higher TMB levels and lower TIDE scores (Figures 7K, L).

### Validation of the prognostic significance of MCM3 in LGG

**FIGURE 8:**
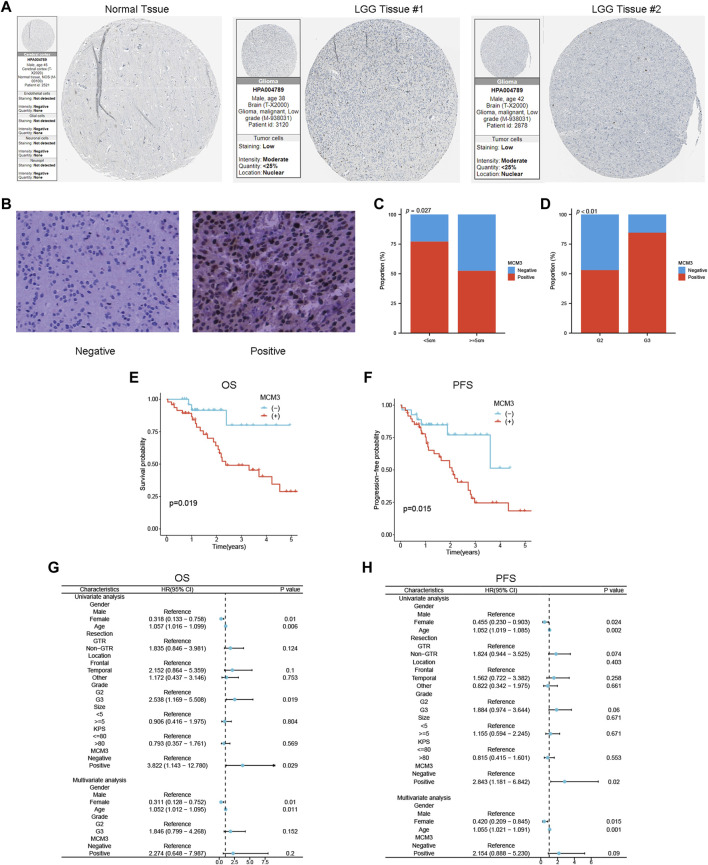
Validation of the association between MCM3 and clinical features and prognosis of LGG. **(A)** MCM3 protein expression in normal and tumor tissues from HPA database. **(B)** Representative plots of negative and positive immunohistochemical results. **(C, D)** Relationship between MCM3 expression and tumor size and grade. **(E, F)** Survival curves for OS and PFS in clinical cohort. **(G, H)** Univariate and multivariate Cox regression analysis for OS and PFS.

IHC results from the HPA showed that MCM3 expression was elevated in LGG tissue (Figure 8A). The clinical features of our validation cohort are shown in Supplementary Table S2. Figure 8B shows a typical representation of negative and positive MCM3 expression in our cohort. By using chi-square tests, we found that the rate of MCM3 positivity was significantly greater in patients with tumours ≥ 5 cm in size and grade III tumours (Figures 8C, D). KM methods showed that MCM3-positive patients had shorter OS and PFS than MCM3-negative patients (Figures 8E, F). In addition, univariate analysis demonstrated that MCM3 expression was correlated with OS and PFS, thus further confirming its prognostic significance in LGG. However, MCM3 did not have independent prognostic significance in our cohort, which may be related to the small sample size (Figures 8G, H).

## Discussion

MCM3, which is a component of the hexameric protein complex, has diagnostic and prognostic value in some cancers (Hsu et al., 2021; Li et al., 2020; Li et al., 2021; Lokkegaard et al., 2021; Zhou et al., 2020). However, the role of MCM3 in other cancer types is unclear. In our study, a pan-cancer analysis and a single-cell analysis were performed to explore the prognostic role, immunological value, and associated biological mechanisms of MCM3. Furthermore, given that MCM3 is closely related to LGG in many ways, we further analysed the relationships between the clinical features, prognosis, and potential biological functions of MCM3 and LGG and validated its prognostic value in a clinical LGG cohort.

Our pan-cancer analysis demonstrated that MCM3, which may have significant prognostic value, was upregulated in 25 tumours, including GBM, LGG, and UCEC. High MCM3 expression was related to poor prognosis in LGG, ACC, LIHC, KICH and SARC patients. Cao et al. suggested that MCM3 may serve as a potential prognostic biomarker for medulloblastoma; this was the first study to elucidate the correlation between MCM3 and central nervous system tumours (L. Cao et al., 2022). Previous studies have shown that MCM2, MCM3 and MCM7 levels are closely linked to glioma prognosis (Söling et al., 2005). Moreover, MCM3 was an independent predictor of prognosis in anaplastic astrocytoma patients (Söling et al., 2005). However, the role of MCM3 in LGG remains unknown. This study was the first to show that high MCM3 expression was linked to shorter OS, DSS, and PFI in LGG patients. Aporowicz’s research suggested that MCM3 could serve as a diagnostic and proliferative marker of ACC (Aporowicz et al., 2019). Our data showed that high MCM3 expression was associated with shorter OS, PFI, and DSS in ACC patients and may be an effective complement for identifying potential markers of ACC. Previous studies have shown that MCM3 is a potential marker for the diagnosis, treatment, and prognosis of LIHC (Zhuang, Yang and Meng, 2018; Yang et al., 2019; Mohamed et al., 2022). Our study showed that high MCM3 expression was associated with shorter OS, PFI, DFI, and DSS in LIHC patients, which was consistent with previous results. A previous study suggested that MCM3 phosphorylation is a new mechanism for regulating the proliferation and apoptosis of renal cell carcinoma cells (Gao et al., 2020). KICH is a type of renal cell carcinoma. Our study showed that high MCM3 levels were associated with shorter OS, PFI, and DSS in KICH patients. Moreover, MCM3 was also closely linked to OS, PFI, and DSS in SARC patients, and this was the first study to show the relationship between MCM3 and SARC patients. Kang et al. reported that high MCM3 expression at both the mRNA and protein levels was associated with longer survival in tubo-ovarian high-grade serous carcinoma patients (Kang et al., 2022). Another study reported that MCM3 is a marker of proliferation in ovarian malignancies (Kobierzycki et al., 2013). In this study, we found that high MCM3 expression in OV was associated with longer PFI, DSS and OS. Therefore, the role of MCM3 in OV should be further evaluated.

We found that in most cancers, MCM3 was closely related to the immune score, stromal score, and ESTIMATE score. In addition, MCM3 was closely associated with tumour-infiltrating immune cells in most cancers. By secreting immunosuppressive cytokines, Tregs downregulate the expression of stimulatory molecules, thus inhibiting the activation of effector T cells and reducing T-cell infiltration in LGG (Lim et al., 2018; Haddad et al., 2022). Ahmadzadeh’s study demonstrated that CD8^+^ T cells stimulate granulocytes to produce granulocyte colony-stimulating factor and perforin to kill tumour cells (Ahmadzadeh et al., 2009). Previous studies have shown that CD4^+^ T cells play an important role in directly eliminating tumours or indirectly providing support for the tumour-killing function of CD8^+^ T cells (Kennedy and Celis, 2008; Melssen and Slingluff, 2017; Borst et al., 2018). In our study, MCM3 was closely associated with CD4^+^ T cells and CD8^+^ T cells in KIRC, LGG, LIHC, PCPG, PRAD and THCA. However, the effect of MCM3 on the immune microenvironment of these cancers and its prognostic value require further study. With the development of high-throughput sequencing technologies, many targets and methods for screening potential beneficiaries of immunotherapy have been identified (Giustini and Bazhenova, 2021; Zhang et al., 2021). In this study, we investigated the potential of MCM3 as a novel predictor of immunotherapy efficacy. The expression of MCM3 was strongly associated with the expression of immune checkpoint molecules and the TMB in most cancers. An earlier study suggested that TMB can be used as a marker of ICB response, with patients with higher TMB levels benefiting more from ICB (Newman et al., 2020). However, McGrail et al. argued that a high TMB does not predict ICB responses in all cancers (McGrail et al., 2021). Overall, MCM3 expression was positively related to TMB in 12 cancers, especially ACC, DLBC, LGG, PAAD and STAD, thus suggesting that patients with high MCM3 expression in these cancers may be more sensitive to immunotherapy. Furthermore, there was a significant difference in the response to immunotherapy between patients with high and low MCM3 in the four clinical cohorts receiving ICB, thus suggesting that MCM3 is a good predictor of immunotherapy response. The TIDE score is another predictor of ICB therapy response, and a low TIDE score is associated with increased sensitivity to ICB therapy (Jiang et al., 2021). In LGG, we found that patients with high MCM3 expression had a greater TMB and a lower TIDE score; therefore, this group of patients could benefit from ICB therapy. Yang et al. identified MCM3 as being a potential therapeutic target for HCC (Yang et al., 2019). Kang et al. showed that MCM3 was associated with immunotherapy in patients with tubo-ovarian high-grade serous carcinoma (Kang et al., 2022). The results of these studies were consistent with our study. The study by Jonathan demonstrated that MSI may be associated with the presence of new immunogenic epitopes to more precisely guide immunotherapy (Dudley et al., 2016). A previous study showed that LUSC patients with high MSI tend to have improved OS (Hu et al., 2023). In our study, MCM3 expression was positively related to MSI level in STAD, KIRC, LUAD and LUSC. Taken together, these findings highlight the potential of MCM3 as being a predictor for immunotherapy efficacy. Drug sensitivity analysis suggested that the expression of MCM3 was positively correlated with sensitivity to 17-AAG, bleomycin (50 µM), RDEA119, trametinib and selumetinib. These data may provide some basis for therapies targeting MCM3.

Tumours are diseases in which cells undergo continual excessive division. Cell cycle checkpoints serve to prevent genetic errors during cell division (Matthews et al., 2022). In our research, single-cell analysis demonstrated that MCM3 was associated with the cell cycle, DNA damage and DNA repair across cancers. In previous studies, MCM3 was shown to act as a proliferation marker and regulate programmed cell death in tumour cells, such as hepatocellular carcinoma, tubo-ovarian high-grade serous carcinoma, oral squamous cell carcinoma, odontogenic cysts and ameloblastoma (Valverde et al., 2018; Jaafari-Ashkavandi et al., 2019). Our study is consistent with the abovementioned findings, thus suggesting that MCM3 may function as a cell cycle checkpoint. Taken together, these results suggest that MCM3 could be not only an immunotherapy target but also a cell cycle checkpoint, thus indicating that MCM3 may be a promising therapeutic target in cancer.

We found that MCM3 expression is correlated with clinical features and prognosis in LGG and is an independent prognostic parameter of LGG. The MCM3-based model can accurately predict the prognosis of LGG, exhibiting good potential for clinical application. GO and KEGG results indicated that MCM3 was mainly involved in cell cycle-related processes and cancer-related pathways. Stewart et al. suggested that MCM3 is involved in the EMT process, thus promoting the invasion and metastasis of prostate cancer (Stewart et al., 2017). Another study demonstrated that MCM3 is overexpressed in medulloblastoma and is involved in tumour cell invasion and metastasis (Lau et al., 2010). Our single-cell and GSEA analyses showed that MCM3 was closely linked to EMT, which to some extent explained the poor prognosis of LGG patients caused by MCM3 overexpression. In addition, previous studies have demonstrated that essential nodes in crucial pathways may be specifically blocked to slow glioma progression, which further suggests that MCM3 may be a promising therapeutic target for LGG (Tang et al., 2014; Liu et al., 2023). In our clinical cohort, MCM3 expression was correlated with the clinical features and prognosis of patients with LGG. However, some limitations of our study should be considered. The MCM3 expression results and prognostic value in most cancers were mainly determined based on publicly available data and need to be validated in clinical cohorts. Moreover, the mechanism by which MCM3 affects the occurrence and development of LGG needs to be clarified by further experiments. Whether MCM3 can predict immunotherapy responses or serve as a novel immunotherapy target needs to be confirmed by additional experimental and clinical trial data.

## Conclusion

Our study suggested that MCM3 can be used as a potential prognostic marker for tumours and may be associated with tumour immunity. In addition, MCM3 is a promising predictor of immunotherapy responses.

## Figures and Tables

**Table:** 

**Pan-cancer**	
**ACC**	Adrenocortical carcinoma
**BLCA**	Bladder Urothelial Carcinoma
**BRCA**	Breast invasive carcinoma
**CESC**	Cervical squamous cell carcinoma and endocervical adenocarcinoma
**CHOL**	Cholangiocarcinoma
**COAD**	Colon adenocarcinoma
**DLBC**	Lymphoid Neoplasm Diffuse Large B-cell Lymphoma
**ESCA**	Esophageal carcinoma
**GBM**	Glioblastoma multiforme
**HNSC**	Head and Neck squamous cell carcinoma
**KICH**	Kidney Chromophobe
**KIRC**	Kidney renal clear cell carcinoma
**KIRP**	Kidney renal papillary cell carcinoma
**LAML**	Acute Myeloid Leukemia
**LGG**	Lower-Grade Glioma
**LIHC**	Liver hepatocellular carcinoma
**LUAD**	Lung adenocarcinoma
**LUSC**	Lung squamous cell carcinoma
**MESO**	Mesothelioma
**OV**	Ovarian serous cystadenocarcinoma
**PAAD**	Pancreatic adenocarcinoma
**PCPG**	Pheochromocytoma and Paraganglioma
**PRAD**	Prostate adenocarcinoma
**READ**	Rectum adenocarcinoma
**SARC**	Sarcoma
**SKCM**	Skin Cutaneous Melanoma
**STAD**	Stomach adenocarcinoma
**TGCT**	Testicular Germ Cell Tumors
**THCA**	Thyroid carcinoma
**THYM**	Thymoma
**UCEC**	Uterine Corpus Endometrial Carcinoma
**UCS**	Uterine Carcinosarcoma
**UVM**	Uveal Melanoma

## Data Availability

The datasets presented in this study can be found in online repositories. The names of the repository/repositories and accession number(s) can be found in the article/Supplementary Material.
