# Supplementary material for: The prognostic and immunological role of MCM3 in pan-cancer and validation of prognosis in a clinical lower-grade glioma cohort
Source: Front Pharmacol. 2024 Apr 18;15:1390615. doi: 10.3389/fphar.2024.1390615 (PMC11063780; doi:10.3389/fphar.2024.1390615)
Supplement: Supplementary file 1 [file Table1.DOCX]

| **Clinicopathological features** |  | **TCGA(n=447)** | **CGGA (n = 420)** |
| --- | --- | --- | --- |
| Age |  |  |  |
| ≤40 |  | 219 | 211 |
| ＞40 |  | 228 | 208 |
| NA |  | 0 | 1 |
| Gender |  |  |  |
| Male |  | 249 | 235 |
| Female |  | 198 | 185 |
| Grade |  |  |  |
| II |  | 213 | 172 |
| III |  | 234 | 248 |
| IDH status |  |  |  |
| Mutant |  | 360 | 288 |
| Wild-type |  | 85 | 94 |
| NA |  | 2 | 38 |
| 1p19q codeletion status |  |  |  |
| Non-codeletion |  | 298 | 257 |
| Codeletion |  | 149 | 125 |
| NA |  | 0 | 38 |
| TCGA: The Cancer Genome Atlas; CGGA: Chinese Glioma Genome Atlas; IDH: isocitrate dehydrogenase. | | | |

**Supplementary Table 1**. Clinicopathological features of TCGA and CGGA cohorts.
