# Supplementary figures and images for: The prognostic and immunological role of MCM3 in pan-cancer and validation of prognosis in a clinical lower-grade glioma cohort
Source: Front Pharmacol. 2024 Apr 18;15:1390615. doi: 10.3389/fphar.2024.1390615 (PMC11063780; doi:10.3389/fphar.2024.1390615)

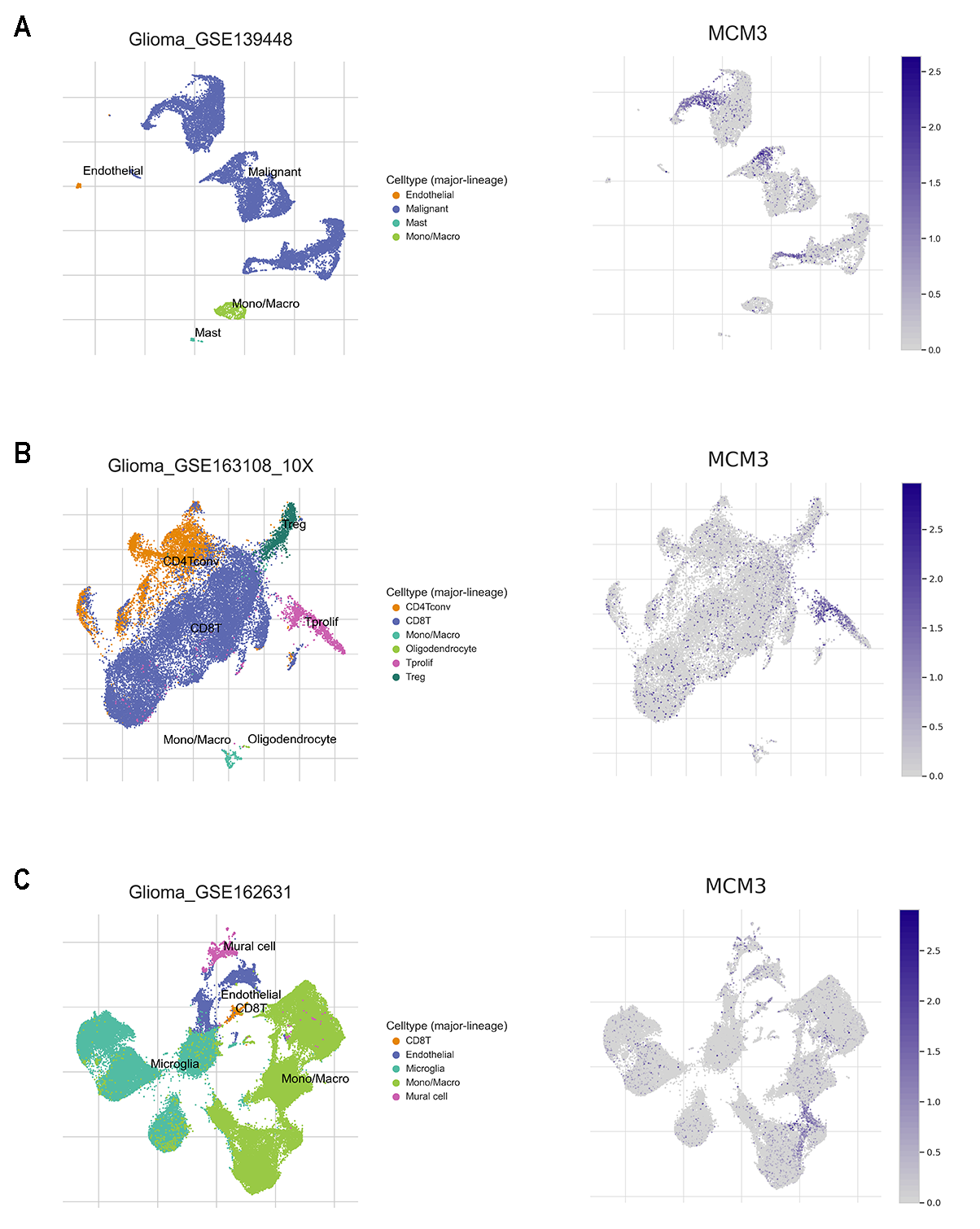

Supplement: Supplementary file 2 [file Image3.TIF]

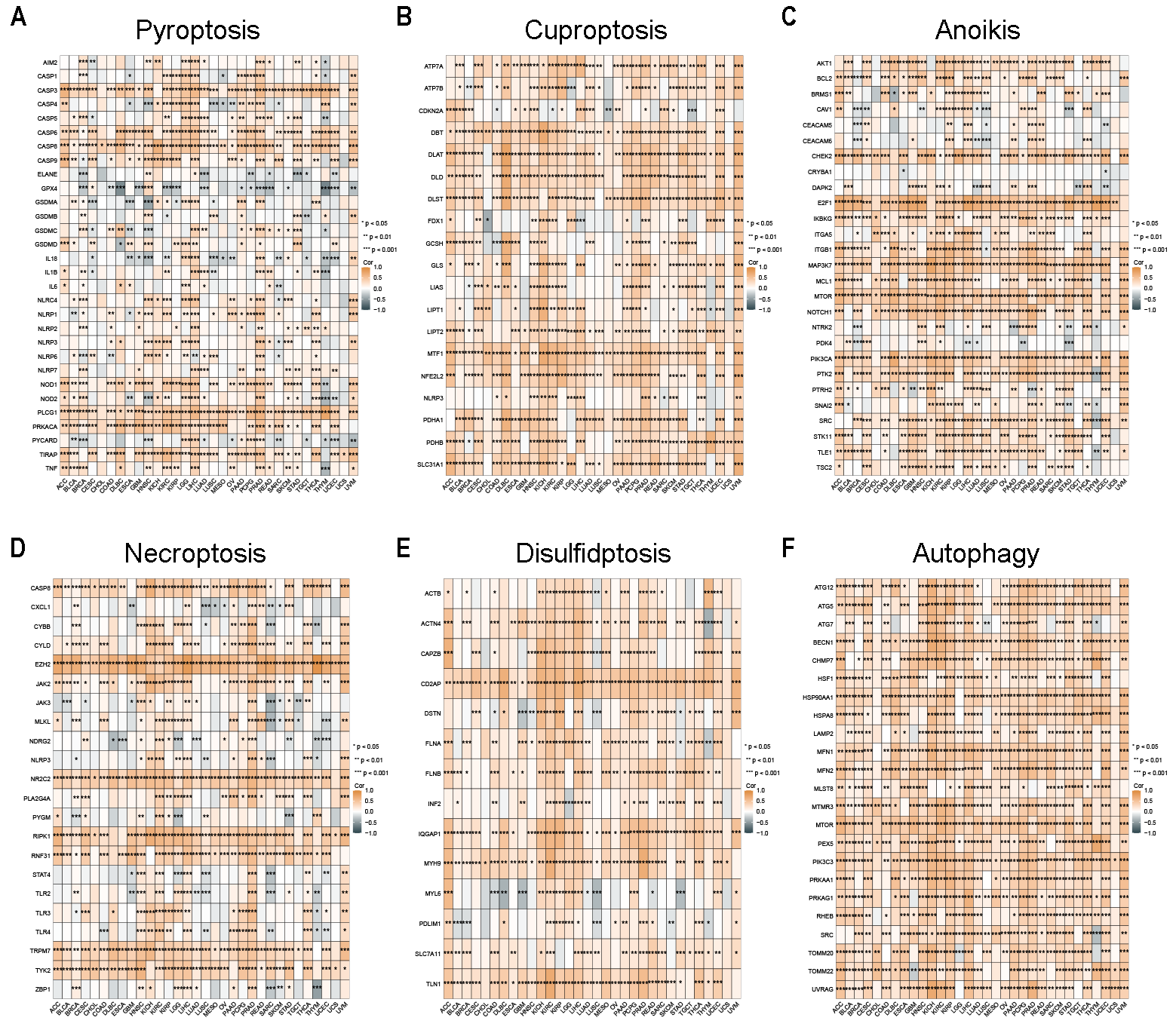

Supplement: Supplementary file 3 [file Image2.TIF]

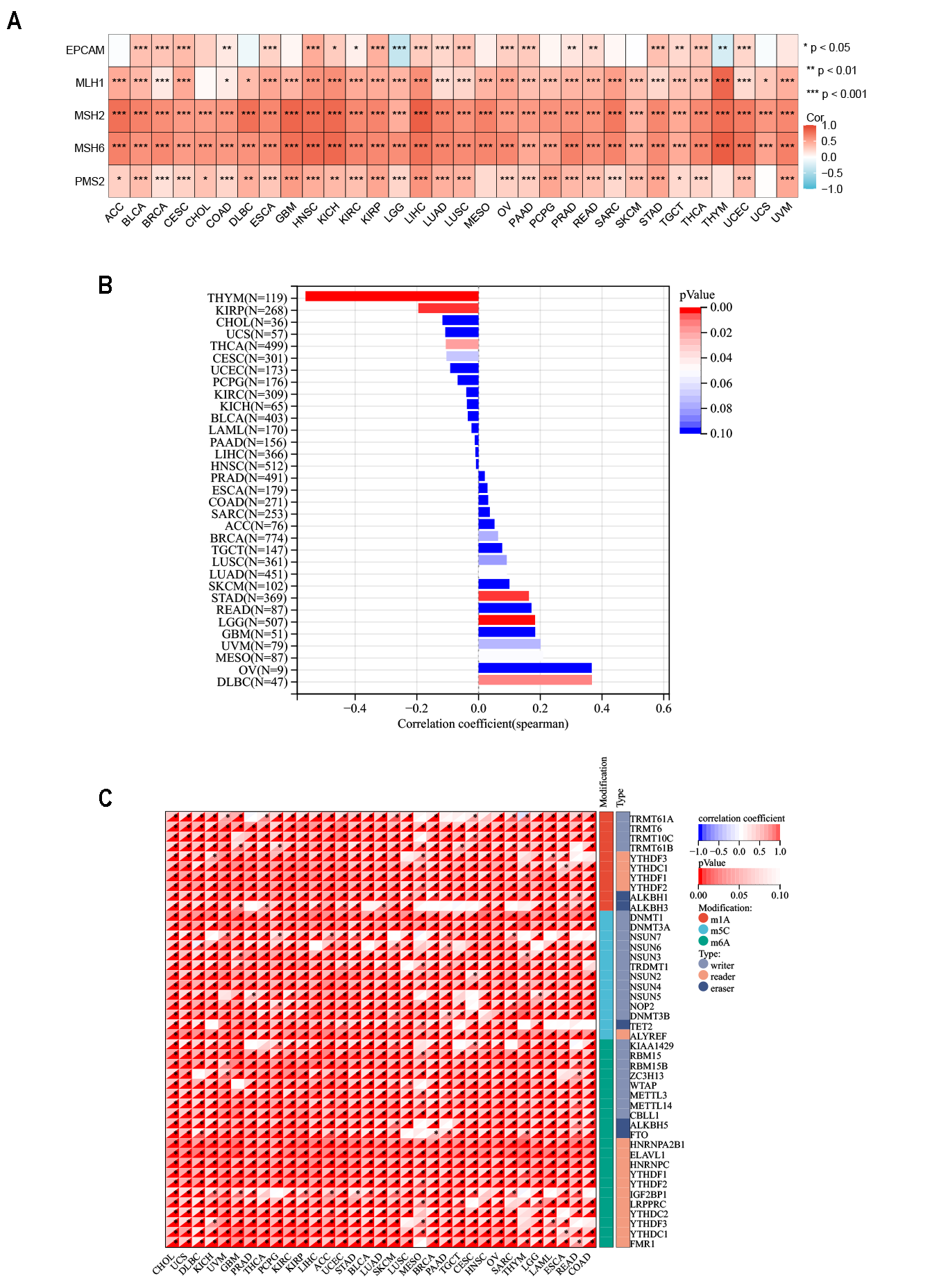

Supplement: Supplementary file 4 [file Image1.TIF]
