# Supplementary material for: The prognostic and immunological role of MCM3 in pan-cancer and validation of prognosis in a clinical lower-grade glioma cohort
Source: Front Pharmacol. 2024 Apr 18;15:1390615. doi: 10.3389/fphar.2024.1390615 (PMC11063780; doi:10.3389/fphar.2024.1390615)
Supplement: Supplementary file 5 [file Table2.DOCX]

**Supplementary Table 2.** Clinical features of 75 patients in validation cohort.

| **Clinical features** | **Number (%)** |
| --- | --- |
| Gender  Male  Female  Age (years)  ≤40  ＞40  Resection extent  GTR  Non-GTR  Location  Frontal  Temporal  Other  Grade  II  III  Size  ＜5  ≥5  KPS  ≤80  ＞80  MCM3  Negative  Positive | 36 (48)  39 (52)  31 (41.3)  44 (58.7)  43 (57.3)  32 (42.7)  43 (57.4)  19 (25.3)  13 (17.3)  49 (65.3)  26 (34.7)  35 (46.7)  40 (53.3)  30 (40)  45 (60)  27 (36)  48 (64) |
| GTR: gross total resection; STR: subtotal resection; KPS: Karnofsky performance status score. | |
